# Growth factor loading on aliphatic polyester scaffolds

**DOI:** 10.1039/d0ra10232f

**Published:** 2021-02-10

**Authors:** Hong Shen, Xixue Hu

**Affiliations:** Beijing National Laboratory for Molecular Sciences, State Key Laboratory of Polymer Physics and Chemistry, Institute of Chemistry, Chinese Academy of Sciences Beijing 100190 China shenhong516@iccas.ac.cn +86-10-62581241; CAS Key Laboratory for Biomedical Effects of Nanomaterials and Nanosafety, National Center for Nanoscience and Technology Beijing 100190 China huxx@nanoctr.cn +86-10-82545676

## Abstract

Cells, scaffolds and growth factors are three elements of tissue engineering. The success of tissue engineering methods relies on precise and dynamic interactions between cells, scaffolds and growth factors. Aliphatic polyester scaffolds are promising tissue engineering scaffolds that possess good mechanical properties, low immunogenicity, non-toxicity, and adjustable degradation rates. How growth factors can be loaded onto/into aliphatic polyester scaffolds and be constantly released with the required bioactivity to regulate cell growth and promote defect tissue repair and regeneration has become the main concern of tissue engineering researchers. In this review, the existing main methods of loading growth factors on aliphatic polyester scaffolds, the release behavior of loaded growth factors and their positive effects on cell, tissue repair and regeneration are introduced. Advantages and shortcomings of each method also are mentioned. It is still a great challenge to control the release of loaded growth factors at a certain time and at a concentration simulating the biological environment of native tissue.

## Abstract 2

Cells, scaffolds and growth factors are three elements of tissue engineering. Growth factors precisely controlled by a scaffold will be used in conjunction with the scaffolds and cells to repair and regenerate defect tissue.

## Graphical abstract

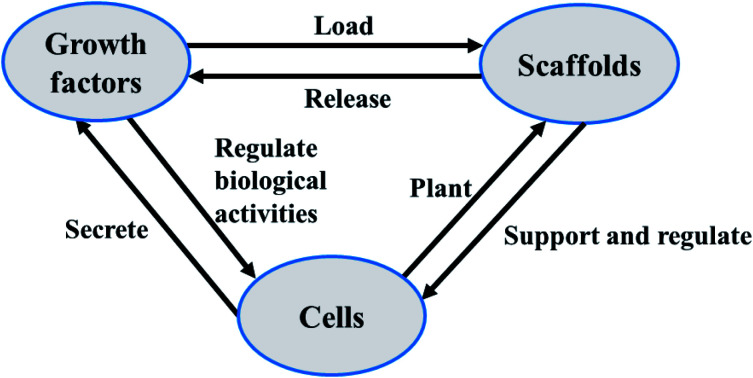

## 1. Introduction

Tissue engineering is an interdisciplinary subject that utilizes the principles of engineering and life science to study and develop bioactive artificial substitutes for the maintenance, restoration, or construction of human damaged tissues.^1,2^ The fundamental goal of tissue engineering is to promote formation and integration of tissue to functional structures or organs. Cells, scaffolds and growth factors are the three elements of tissue engineering.^3,4^ The success of tissue engineering methods relies on the precise and dynamic interactions between cells, scaffolds and growth factors.^5,6^

Scaffolds provide sufficient substrate for cells adhesion and growth, define the growth space of the tissue in the body, and connect the new tissue into one piece. Scaffolds structurally enhance the defect gap, maintain the integrity of the tissue structure and prevent deformation. Moreover scaffolds provide a place for cells to obtain nutrients, exchange gases, and excrete waste. The scaffolds can gradually degrade and disappear as the cells proliferate to make space for new tissue. Ideal scaffolds must successfully be loaded some growth factors to induce cell differentiation into the desired tissue. Therefore, the scaffold not only provides physical support for the cell in tissue engineering, but also acts as an extracellular matrix (ECM) to regulate cell proliferation, differentiation and morphogenesis.^7,8^

Growth factors play critical roles in regulating cell's adhesive, migration, proliferation, differentiation, gene expression, maturation and apoptosis.^9–11^ Tissue regeneration with only cells and scaffolds is often unsuccessful. In this case, exogenous growth factors must be in place to initiate the regeneration process. Different types of cells often require different growth factors to promote their growth. Damaged tissue also needs some growth factors to promote its repair. However, the bioactivity of growth factors cannot always be expected when they are injected into the body in a soluble form, because of their short duration of retention at wound sites and short half-life caused by susceptibility to enzymatic and thermal degradation *in vivo*.^10,12,13^ If growth factors are embedded or fixed on the scaffolds, the scaffolds can prevent the growth factor from direct contacting with water, limit their diffusion and thus prolong the activity *in vivo*. Therefore, the ideal scaffolds should have the function of secreting and slow releasing growth factors in the amount of natural tissue required during clinical application which getting to the purpose of building the biological model like natural tissue. Thus, the function simulation of the scaffolds is realized.

Scaffold materials used in tissue engineering should be biocompatible, supporting tissue cell growth, inducing tissue regeneration and biodegradable. Scaffold materials mainly include inorganic materials, natural polymer materials and synthetic polymer materials.^14–22^ Inorganic materials such as tricalcium phosphate, hydroxyapatite, coral and so on, which are generally only suitable for bone tissue engineering, are difficult to process and have high brittleness.^14,15^ Natural polymer materials such as collagen, hyaluronic acid, chitosan and so on have uncontrollable degradation rate *in vivo*, poor strength and processing performance and bad batch repeatability.^19^ Synthetic polymer materials such as polyhydroxyalkanoates (PHAs), aliphatic polyesters and polyurethanes (PUs) can be mass produced by chemical synthesis with controlled properties and good repeatability. However, the application of PHAs in tissue engineering scaffolds is limited by the disadvantages such as low degradation rate, high brittleness and contamination by pyrogenic compounds.^20^ Degradation products of PUs and their corresponding toxicity levels still is unclear.^21^ Recently, aliphatic polyester biodegradable polymers, such as polylactide (PLA), polyglycolide (PGA), poly(lactide-*co*-glycolide) (PLGA) and polycaprolactone (PCL), have been extensively researched as scaffold materials for tissue engineering due to their good mechanical property, low immunogenicity, non-toxicity, and adjustable degradation rate.^23–27^ So, it becomes an issue of concern for tissue engineering researchers that how to load a growth factor onto the aliphatic polyester scaffold and release it at a certain time and concentration to promote cell growth and differentiation. To the best of our knowledge, no review articles have systematically overviewed growth factor loading methods on aliphatic polyester scaffolds and their corresponding release behavior. In this review, we detail the existing methods of loading growth factors on aliphatic polyester scaffolds and the effects released growth factors on cell growth and tissue generation. Moreover, problems of different loading methods also are mentioned.

## 2. Loading methods of growth factors on aliphatic polyester scaffolds

### 2.1 Direct blending or soaking method

**Fig. 1:**
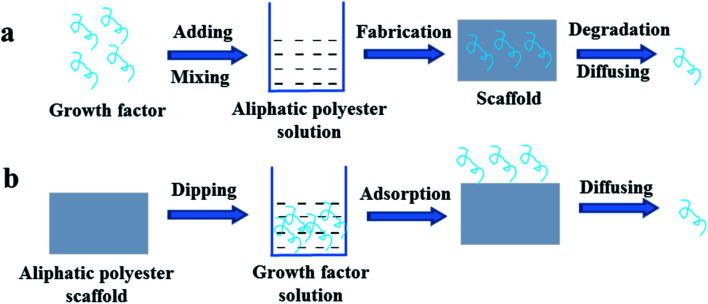
Schemes of growth factor loading on aliphatic polyester scaffolds by directly blending (a) or soaking (b) method and releasing loaded growth factor.

Growth factors can be incorporated directly into the various aliphatic polyester scaffolds including microsphere, gel, foam and membrane during the scaffold fabrication or immobilized on the surface by soaking the scaffold in growth factors solution after fabrication.^28–39^ Growth factors mix with the polymer solution, and then the embedded growth factor scaffolds are prepared by solution casting, phase separation, electrospinning and double emulsion methods, *et al.* The embedded growth factor is gradually released with diffusion and degradation of polymer, as shown in Fig. 1a. By direct mixing the growth factors with the polymer solution, one or more of the growth factors can be embedded in the whole or different parts of the aliphatic polyester scaffold at the same time. The release kinetics of growth factors mainly depend on the types of aliphatic polyester, not the types of growth factors, *i.e.* degradation rate of the polymer mainly determines the release patterns of growth factors. Sahoo *et al.* encapsulated basic fibroblast growth factor (bFGF) in two types of PLGA nanofiber scaffolds using blending and electrospinning (Group I) and coaxial electrospinning (Group II). bFGF randomly dispersed in Group I and distributed as a central core within Group II nanofibers. bFGF encapsulation efficiency in the both scaffold groups was 54 ± 5%. The scaffolds in Group I released all the encapsulated bFGF in 7 days and the scaffolds in Group II could sustain the release till 14 days, which was attributed to a combination of passive diffusion across nanopores on the nanofiber surface and degradation of the nanofibers. bFGF encapsulated in the Group I scaffolds was more conducive for fibroblastic differentiation of bone marrow stem cells (BMSCs).^34^ Schofer *et al.* further demonstrated bone morphogenetic protein-2 (BMP-2) incorporated into poly-l-lactic acid (PLLA) nanofiber scaffolds by the blending and electrospinning enhanced bone healing *in vivo*.^35^ Hong *et al.* fabricated multilayered fibrous scaffold capable of controlling the release of multiple growth factors.^32^ The neurotrophin (NT-3) and brain-derived neurotrophic factor (BDNF) loaded into the PLGA 6535 nanofiber layer released faster than the platelet-derived growth factor (PDGF) loaded into the PLGA 8515 nanofiber layer, in which the ratio of lactide (LA) and glycolide (GA) is 65 : 35 and 85 : 15 respectively. The release of NT-3 and BDNF from PLGA 6535 reached a plateau after six weeks, while the PDGF from PLGA 8515 began to exhibit a plateau after eight weeks. The fast release of NT-3 and BDNF as well as the slow release of PDGF gave the best results in terms of nerve. Similarly, Zhou *et al.* prepared bilayer scaffolds consisting of PDGF-incorporated PLGA 75/25 nanofibers and vascular endothelial growth factor (VEGF)-incorporated PLGA 50/50 nanofibers with orthogonal fiber orientations by a sequential emulsion electrospinning process. The encapsulation efficiencies of VEGF and PDGF were 57.27 ± 5.04% and 51.76 ± 2.37%, respectively. The release of VEGF from the PLGA 50/50 layer was faster than that of PDGF from the PLGA 75/25 layer, which should mainly arise from the relatively faster degradation rate of PLGA 50/50 than that of PLGA 75/25. In the sustained release stage, the release of VEGF and PDGF from different nanofibers follows a complex diffusion-and-erosion-controlled release model. The bilayer scaffolds show appropriate structural and biochemical characteristics to effectively direct and stimulate the behaviors and functions of human vascular endothelial cells (HUVEC) and smooth muscle cells (vSMC).^36^

Although the growth factors of direct blending in the scaffolds have certain positive effect on the cell growth and tissue regeneration, the direct blending method is limited. Growth factor is generally water-soluble and aliphatic polyester polymer is oil-soluble. When the scaffold is prepared, mixing the aqueous solution of growth factor into the polymer solution may be uneven, resulting in the uneven distribution of growth factor in the scaffold. Moreover, the organic solvent also leads to inactivation of growth factors.^37–39^ In the preparation of salt or sugar-induced porous scaffold, the process of washing will also lead to loss of growth factors. To avoid the influence of organic solvent on bioactivity of growth factors organic solvent-free direct incorporating growth factors into the aliphatic polyester scaffolds are developed.^37–39^ Murahashi *et al.* reported multi-layered PLLA nanosheets loaded with recombinant human fibroblast growth factor-2 (rhFGF-2) that was incorporated into the nanosheets by dropping onto the middle of the PLLA nanosheet. Subcutaneous implantation revealed that the spread of rhFGF-2 was limited in the tri-layered nanosheets. The multi-layered PLLA nanosheets loaded with rhFGF-2 displayed sustained release effectively enhanced bone regeneration in mouse femoral bone defects.^37^ Diaz-Gomez *et al.* prepared porous solid poly(ε-caprolactone) (PCL) scaffolds incorporating preparation rich in growth factors (PRGF) and PCL/PLGA composite scaffolds incorporating platelet-rich plasma (lPRP) using a solvent-free foaming method based on supercritical fluid technology without post-processing steps.^38,39^ The processing method allowed a uniform distribution of the growth factors in the scaffold and retained activity. Incorporation of pregelified starch in the scaffold adjusted the polymer-growth factor interaction. Growth factor release was sustained and governed by diffusion mechanism in the 7 day period tested. PRGF and lPRP in these scaffolds increased human adipose-derived MSCs attachment and proliferation.

On the other hand, after aliphatic polyester scaffold is formed, the scaffold is soaked in the solution of growth factors. In result, the growth factors are adsorbed on the surface of the scaffold and they are released by diffusion (Fig. 1b). Scaffold is directly immersed in growth factor solution, and then the growth factor is loaded on the scaffold mainly by physical adsorption that lead to the rapid release of loaded growth factors. The loading amount is related to the topology of the scaffold surface. The loading efficiency of growth factors on the scaffolds with smooth and micron topological surface is very low, so the directly soaking method hardly is used.^29,40^ Comparing with smooth and micron topological scaffolds, scaffolds with nano topological structure are more conducive to loading growth factor. Xia *et al.* prepared PLLA nanofibrous scaffold loaded VEGF on the surface by direct soaking and recombinant human nerve growth factor (NGF) in the core by direct blending, respectively.^33^ VEFG was released in the first few days but the NGF could be continuously released for more than 1 month. The scaffold loaded VEGF and NGF could enhance the neural differentiation of induced pluripotent stem cells-derived neural crest stem cells (iPSCs-NCSCs) *in vitro* and improve neovascularization as well as nerve healing *in vivo*. However the poor hydrophilicity and lack of functional group of the aliphatic polyester polymers often result in low loading efficiency of growth factor by solution dipping method and burst release of loaded growth factor.^40^ So, the application of direct soaking method is greatly limited and thus hardly reported.

### 2.2 Surface coating combined with covalent binding method

**Fig. 2:**
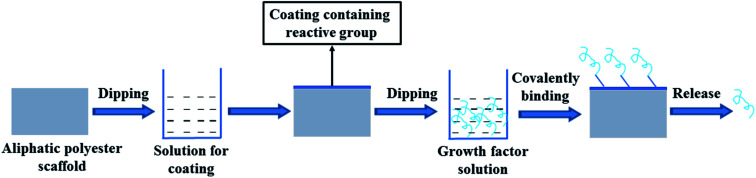
Scheme of growth factor loading on aliphatic polyester scaffolds by surface coating combined with covalent binding method and releasing loaded growth factor.

Theoretically, growth factors may be fixed on a scaffold by covalent binding to the active group on the surface of the aliphatic polyester scaffold. Since lack of functional groups in the backbone of the polymer, it is difficult directly covalent binding growth factors on the surface of scaffold. It is a feasible method that first introducing a coating containing reactive groups on the scaffold surface and then growth factors are loaded on the scaffold by covalent binding between growth factors and reactive groups (Fig. 2).

In order to increase the amount of active groups, Zeng *et al.* coated polypyrrole on PLLA fibers by oxidation polymerization and then poly-l-lysine (PLys) was coated on the surface of PPy-PLLA fibers by ethyl-3-[3-(dimethylamino)propyl] carbodiimide hydrochloride (EDC) chemistry.^41^ NGF could be conjugated with the PLys, so it was easy to quantitatively control the amount of conjugated-NGF on the PLLA fibers by controlling the amount of PLys. The PLLA fibers conjugated NGF could support PC12 cells neurite outgrowth and axon elongation. Niger *et al.* first coated collagen on the PLLA nanofiber scaffold and then cross-linked transforming growth factor-beta 3 (TGF-β3) to collagen-coated PLLA scaffold by transglutaminase (TG2) enzyme catalyzing the formation of covalent N-ε-(c-glutamyl)lysine amide bonds between individual protein strands to form a permanent network of polypeptides.^42^ TGF-β3 irreversibly cross-linked by TG2 to collagen PLLA scaffolds retained its biological activity and was capable of inducing chondrogenic differentiation of hBMSCs.

Polydopamine (PDA) can be readily obtained from dopamine under an alkaline conditions by spontaneous oxidative polymerization, which can coat on any surface irrespective of their composition, size, and shape. Many drugs and biomolecules carrying thiol and amine groups are easily bound to the surface of the PDA-coated materials by the chemical and physical reaction with catechols of PDA.^43–47^ Some studies have verified that growth factors such as BMP-2, bFGF, insulin-like growth factor 1 (IGF-1) can be effectively bound on the surface of aliphatic polyester scaffold using PDA coating mainly *via* covalent bond and possible hydrogen bond.^48–54^

Surface modification of PDA coating is a simple one-step method that does not need special facilities and maintains the structure of the scaffold material. Furthermore, PDA coating can improve the hydrophilic nature of polylactide scaffolds. It is reported that PDA can significantly promote the adhesion and proliferation of cells. The surface modification by PDA layer can more efficiently immobilize growth factors on the scaffold surfaces than physical adsorption, and the immobilized growth factor is released slowly and steadily from the scaffold in a sustained manner. Moreover, the released growth factors produce positive effects on the seed cells and tissue generation. Zhang *et al.* simultaneously immobilized BMP-2 and IGF-1 on the three-dimensional porous poly(l-lactic-*co*-glycolic acid)/hydroxyapatite (PLGA/HA) scaffold *via* a PDA layer surface modification.^51^ The efficiency of IGF-1 and BMP-2 immobilization on PDA-PLGA/HA scaffolds was approximately 2.5 and 2.7 times more than that on un-coated scaffolds, respectively. The released IGF-1 and BMP-2 exhibited similar release behaviors. After a burst release the release subsequently slowed down with approximately 27% (IGF-1) and 39% (BMP-2) of the total growth factors released after 21 d, respectively. The dual delivery of BMP-2 and IGF-1 *via* PDA coating saved excellent bioactivities for supporting the adhesion, proliferation, and osteogenic differentiation of cell and rabbit radius defect repair. BMP-2 was also immobilized on the 3D-printed poly(lactic acid) (PLA) scaffolds with interconnected microporous architectures *via* the actions of PDA coatings.^52^ The amounts of BMP-2 loaded on the surface of the PLA scaffold was proportional to the concentration of BMP-2. After an initial burst release of BMP-2 during the first 48 h *via* desorption from scaffold surface, BMP-2/PDA/PLA scaffolds exhibited continuous release of BMP-2 for up to 35 days, which mainly attributed to the covalent binding between BMP-2 and PDA. Alkaline phosphatase (ALP) activity and osteocalcin of hMSCs cultured on BMP-2/PDA/PLA were significantly higher compared with PLA and PDA/PLA scaffolds. Lee *et al.* immobilized VEGF on the outside surface of 3D bio-tubular PCL scaffolds by PDA-mediated method.^53^ The immobilization efficiency of VEGF and hydrophilicity of scaffold were significantly enhanced by PDA coating, which markedly enhanced angiogenic differentiation both *in vitro* and *in vivo*. By PDA-mediated method growth factor almost may be immobilized on any structure aliphatic polyester scaffolds, because PDA surface coating doesn't destroy structure of scaffolds and induce degradation of scaffolds. However, the PDA coating can be uneven because of the poor hydrophilicity of aliphatic polyester scaffold and it is difficult to coat the inner surface of long tube and thick porous scaffold.

The surface coating combined with covalent binding method is a useful tool for delivering growth factor in a spatially controlled manner and PDA coating is widely used for loading growth factors on the aliphatic polyester scaffolds. However, other coating covalent binding growth factors are rarely used, since other coating containing reactive group may be difficult to obtain and easy to peel off. The surface coating combined with covalent binding method can lead to conformational disturbances of growth factors, which can significantly decrease their biological activity.^55^ Moreover, the release mechanism of the covalent binding growth factors is still unclear.

### 2.3 Micro-nano particle embedding method

**Fig. 3:**
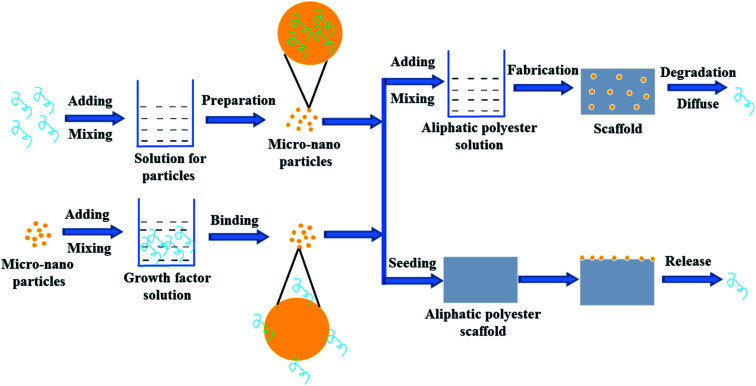
Scheme of growth factor loading on aliphatic polyester scaffolds by micro-nano particle embedding method and releasing loaded growth factor.

In order to control the release rate of the growth factors in the scaffold, the growth factors are first loaded onto/into micro-nanoparticles and then the loaded growth factor particles are directly seeded and fixed on the formed aliphatic polyester scaffolds or mix with polymer to prepare scaffolds with different shapes (Fig. 3). The method of loading growth factors on/in the scaffolds significantly reduce the burst effect. The release rate of growth factors is mainly determined by the properties of micro-nano particles and scaffold. Micro-nano particle materials include natural and synthetic materials such as collagen, dextran, chitosan, gelatin, silica, PLGA, *etc.*^56–65^

Natural biomaterials usually have good biocompatibility, hydrophilicity, and biodegradability. Biomaterials with good biocompatibility can be readily recognized and tolerated by native body.^66^ Growth factors are usually water-soluble and they can be encapsulated into the hydrophilic biomaterial micro-nanoparticles by aqueous method without any contact with the water/oil interface, organic solvents or polymers to avoid loss of bioactivity.^62^ Biodegradability meets the requirements of scaffold degradation with new tissue formation. Therefore natural biomaterials can be used to prepare micro-nano particle for delivering factors. On the other hand, electrospun fibrous membranes are widely-used scaffolds but growth factors are highly susceptible to losing their bioactivity during the process of emulsion electrospinning to incorporate growth factor. In the study of Liu *et al.*, pre-formulated dextran glassy nanoparticles (DGNs) loaded with basic fibroblast growth factor (bFGF) were electrospun into a PLLA fiber.^58^ By the method, the encapsulation efficiency of bFGF for the PLLA electrospun fibrous membrane reached about 48.71%. The encapsulated bFGF could release sustainably nearly 30 days and the bioactivity of bFGF was better than that of encapsulated in the PLLA fibrous membranes by emulsion electrospun. The released bFGF enhanced cell proliferation and intrinsic tendon healing. Similarly, in Tang's study, to release VEGF in a sustained manner with the degradation of PLGA and maintain its bioactivity concurrently, dextran nanoparticles (DNPs) loaded with vascular endothelial growth factor 165 (VEGF165) were pre-formulated by dual-aqueous phase separation method and then electrospun into the PLGA polymer fiber membrane.^62^ The prepared VEGF/DNPs-PLGA membrane was sandwiched by dual-layer SIS to construct a SIS-DNPs/VEGF-PLGA-SIS (SVDPS) composite scaffold, which significantly promoted early therapeutic neovascularization within 2 weeks post-surgery.

However, hydrophilic biomaterials lack the ability to impose a significant barrier against diffusion of embedded growth factors.^67^ Moreover, bad batch repeatability of natural biomaterials also limits widespread adoption and reproducibility of this approach.^68^ Therefore, growth factors are delivered by synthetic biomaterial with controlled properties and good repeatability.^67^ Since PLGA offers a wide range of tunable characteristics such as intrinsic viscosity, rate of degradation and hydrophobicity, growth factors are embedded in PLGA micro-nano particles and then incorporated into other aliphatic polyester scaffolds to control the release of growth factors.^56,57,60,64,65^ Wei *et al.* respectively incorporated PLGA microspheres loaded PDGF-BB and the ones loaded rhBMP-7 into PLLA nano-fibrous scaffolds using a post-seeding method.^56,57^ Sustained release of the growth factors from several days to months was achieved by controlling degradation rate of microspheres on scaffolds. The microsphere-scaffold system was capable of releasing bioactive PDGF-BB and rhBMP-7 in a temporally controlled fashion with prolonged duration. Released PDGF-BB possessed biological activity and rhBMP-7 induced significant ectopic bone formation throughout the scaffold. Tarafder *et al.* first fabricated PLGA microspheres encapsulated with CTGF, TGF-β3, and BMP-2, and then got PCL scaffolds embedded with the above microspheres by layer-by-layer deposition technique using 3D Bioplotter.^60^ PLGA microspheres were able to maintain original structure and protect bioactivities of growth factors. Micro-precise spatial control of multiple growth factors was achieved by interchanging dispensing cartridges during a single printing process. Growth factors loaded in PCL scaffolds *via* PLGA microspheres embedding were released up to 42 days in a sustained manner. The spatially controlled delivery of growth factors in 3D printed scaffolds could guide regeneration of inhomogeneous tissues and multi-tissue complex.

Growth factors are first encapsulated in micro-nanoparticles and then seeded onto or embedded into aliphatic polyester scaffolds, which is the common method to control release of growth factor from scaffolds. However, the biological activity and loading efficiency of growth factors are limited by materials and preparation process of micro-nano particles. In the process of particles or scaffolds preparation, organic solvents may destroy the biological activity of growth factors and reduce loading efficiency of growth factors on/in the micro-nanoparticles.^62^

### 2.4 Plasma treatment combined with growth factor anchoring method

Since lack of reactive group of the aliphatic polyester scaffold, it is difficult to introduce functional groups by common chemical modified method. However, some specific elements or functional groups can be easily introduced onto surface of a scaffold only by selecting and applying some suitable gas under plasma treatment, while it has little effect on bulk properties of the material.^63,69–72^ Since these specific functional groups such as amino, carbonyl, carboxyl and hydroxyl, can provide special chemical reactivity and vary physical properties of the surface, it is benefit to surface functionalization using bioactive molecules.^63,73–76^

**Fig. 4:**
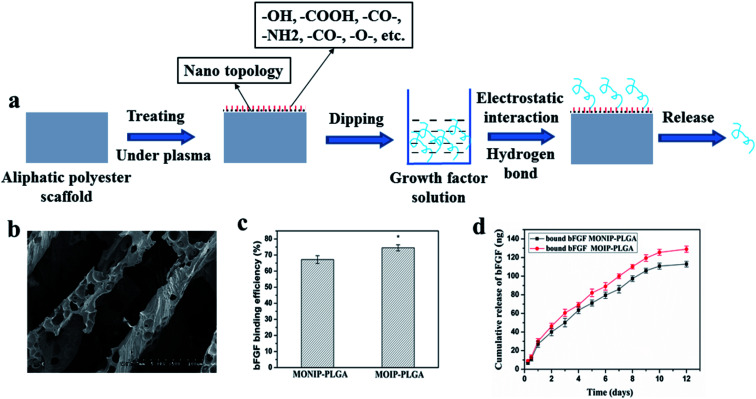
Scheme of growth factor loading on aliphatic polyester scaffolds by plasma treatment combined with growth factor anchoring method and releasing loaded growth factor (a), morphology of MOIP-PLGA scaffold (b), bFGF binding on the plasma-treated MOIP-PLGA scaffold (c) and release profile of binding bFGF (d).^78^

Fig. 4a shows the process of plasma treatment combined with growth factor anchoring method. Nano topology and rich functional groups on the surface of plasma-treated aliphatic polyester scaffolds lead to effective binding of growth factor on the scaffolds by electrostatic interaction and hydrogen bonding. Growth factor release is slowed down and possibly controlled by a thermodynamic equilibrium between the growth factor–scaffold complexes and free growth factor in release medium. By selecting plasma treating parameters, we successfully immobilized bFGF and rhBMP-2 on PLGA scaffolds in a concentration dependent mode.^40,77,78^ The bFGF bound on the plasma-treated PLGA (PT-PLGA) film could maintain bioactivity and be slowly released for 7 days *in vitro*.^40^ Similarly, bFGF was efficiently bound on plasma-treated 3D microtubule-orientated PLGA scaffold with interconnected pores scaffold (MOIP-PLGA) (Fig. 4b and c) and the binding bFGF could slowly release for 10 days *in vitro* keeping bioactivity (Fig. 4d).^78^ The bFGF loaded MOIP-PLGA scaffolds guided vSMCs to better grow along the microtubule direction. Furthermore, immobilization of bFGF on the PT-PLGA scaffold improved adhesion and growth of cells on the PLGA scaffold. rhBMP-2 could be effectively bound onto surface of the oxygen plasma-treated PLGA (OT-PLGA) matrix.^77^ The amount of immobilized rhBMP-2 closely depended on the extent of the improved hydrophilicity and rich polar O-containing groups of the OT-PLGA scaffold. The immobilized rhBMP-2 on PLGA scaffold constantly was released with bioactivity, which stimulated the differentiation of OCT-1 cell and accelerated the process of mineralization of OCT-1 cell in a dose-dependent manner.

The plasma treatment combined with growth factor anchoring method may be hoped to extend to various growth factors and aliphatic polyester polymeric scaffolds by choosing suitable plasma treating parameters. It is an effective method for loading growth factor on aliphatic polyester scaffold and controlling the release of growth factor from the PLGA scaffolds. The loading and release of growth factors can be controlled in certain space by the plasma treatment combined with growth factor anchoring method. Moreover, the method is rapid, clean and without organic solvent pollution. However, the binding growth factors by the method are only on the scaffold surface, which limits long-term continual release of growth factors keeping bioactivity. The plasma treatment combined with growth factor anchoring method is difficult to immobilize growth factors on the inner pore surface of the thicker porous scaffolds and longer tubular scaffolds because of straight irradiation and weaker *trans*-permeability of the plasma ray. If high power and long treatment time are administrated, the outer surface of the scaffolds will deform and degrade seriously.^79^

### 2.5 Heparin-mediated method

Heparin is a highly sulfated macromolecular polysaccharide that can associate with the cell surface and it is one component of extracellular matrix.^80,81^ It is well accepted that the specific electrostatic interactions can occur between the negatively charged sulfate groups of heparin and positively charged amino acid residues of proteins. The electrostatic interaction can enhance binding affinity of the heparin for many growth factors such as VEGF, TGF-β, PDGF, NGF, bone morphogenetic proteins (BMPs) and enable the growth factors to diffuse out in a sustained manner.^82–87^

**Fig. 5:**
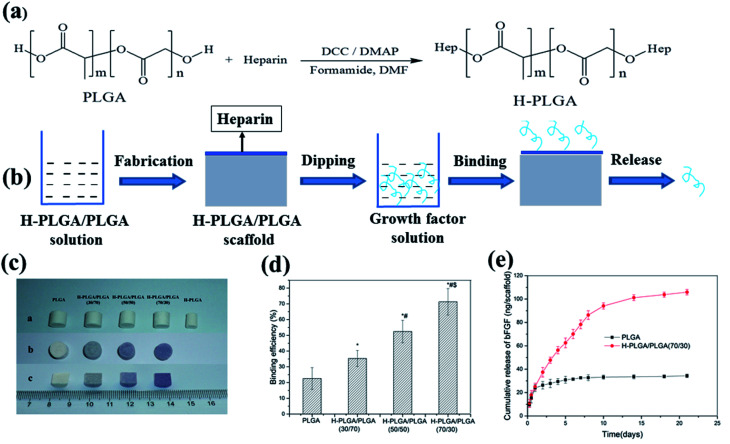
Scheme of growth factor loading on aliphatic polyester scaffolds by H-PLGA and releasing loaded growth factor (a and b), H-PLGA/PLGA scaffolds before and after toluidine blue staining (c), bFGF binding on H-PLGA/PLGA scaffolds (d) and release of binding bFGF (e).^89^

Heparin can be introduced onto the aliphatic polyester scaffolds by physical sorption, ion reaction and covalent binding. Kim *et al.* first coated PLGA/PLLA microfiber scaffolds with human fibroblast-derived matrix (hFDM) and then heparin was conjugated on surface of polymeric scaffolds *via* EDC/NHS chemistry between carboxyl of heparin and amine groups of hFDM to immobilize TGF-β1.^88^ The heparin-grafted hFDM reserved significantly higher amount of TGF-β1 than the simple hFDM. The immobilized TGF-β1 showed a continuous release for 4 weeks after a moderate initial burst release. hFDM/TGF-β1-coated PLGA/PLLA microfiber scaffolds with UCB-MSCs showed effective cartilage healing potential during 12 weeks of *in vivo* implantation into the rabbit knee articular cartilage defects. Sometimes stability of heparin bound on the polymeric scaffolds by physical sorption and ion reaction could not meet the application demand. Among three of them, the directly covalent binding heparin to the polymeric scaffold is the most stable, but it is difficult to directly conjugate large number of heparin with the polymeric scaffolds by chemical method since there are very few functional groups (only two end groups) in backbone of the polymers, especially in the case of using high molecular weight polymers. We reported a heparin-conjugated PLGA (H-PLGA) that was synthesized by reaction of heparin and a low molecular weight PLGA (Fig. 5a).^89^ The heparin-containing PLGA (H-PLGA/PLGA) scaffold was fabricated by blending the H-PLGA with a high molecular weight PLGA. Then bFGF was immobilized on the H-PLGA/PLGA scaffold mainly by static electricity action between them (Fig. 5b and c). The bFGF binding efficiency of H-PLGA/PLGA scaffolds was higher than that of the PLGA scaffold, and increased from 35.3 to 71.3% with changing the content of H-PLGA from 30 to 70% (Fig. 5d). The bound bFGF released *in vitro* slowly from the H-PLGA/PLGA scaffolds and last over two weeks (Fig. 5e). The released bFGF preserved its bioactivity and enhanced the attachment and growth of mouse 3T3 fibroblasts on the H-PLGA/PLGA scaffolds. The H-PLGA mediated method for immobilizing growth factor on aliphatic polyester scaffold is not limited by shape and size of scaffold and growth factor uniformly distributes on the scaffold. However, the binding growth factors by heparin-mediated method are only on the scaffold surface, which limits long-term continual release of growth factors keeping bioactivity and only is appropriate for the early stages of tissue repair. Moreover, it is still required to verify whether the growth factors are released in a free form or as complexes with heparin for heparin-mediated method, which can influence bioactivity of growth factor.^90^

### 2.6 Combination of multiple methods

In order to obtain release behavior matched tissue generation, growth factors are loaded on the aliphatic polyester scaffolds by combination of multiple methods and thus the release of growth factors is also controlled by multiple ways.^91–94^ Li *et al.* fabricated a nanofiber microsphere scaffold by loading hierarchical VEGF.^91^ In this scaffold, VEGF bonded with heparin was encapsulated in heparin conjugated gelatin nanospheres and then further immobilized in the nanofibers of an injectable scaffold of PLLA microspheres. The release of VEGF was controlled by a multiple manner including heparin binding, degradation of the heparin-conjugated gelatin nanosphere and the physical adsorption of the nanofibers. Simultaneous binding VEGF to heparin and encapsulating VEGF into nanospheres significantly decreased the initial burst release of the protein from the microspheres. VEGF was released consistently at a rate of approximately 1–2% per day for the last 3 weeks. This hierarchical microsphere system not only protected the VEGF from denaturation and degradation, but also provided excellent control of its sustained release. The PLLA microsphere scaffolds loaded VEGF promoted the regeneration of pulp-like tissues and a large number of blood vessels.

Tissue regeneration and repair are inseparable from the interaction of multiple growth factors. Different growth factors are simultaneously loaded in/on the scaffolds by combination of multiple methods, which lead to similar or different release behavior. For example, it has been confirmed that many growth factors such as TGF, BMP, IGF-1 and FGF have obvious regulatory effects on cartilage growth. Crecente-Campo *et al.* fabricated a PLGA porous scaffolds loaded BMP-7 and TGF-β3 by encapsulation nanocomplex of cationic PEG derivative coating the complex of heparin, BMP-7 and TGF-β3.^92^ Release of BMP-7 and TGF-β3 was controlled by a multiple-layer manner including heparin binding, cationic PEG derivative binding, composition and structure of scaffold. The release of both growth factors had no burst effect and an almost two-week release lag-time for TGF-β3 was observed. Both growth factors were released with near zero-order kinetics and were able to provide at least 27 days of sustained release. The controlled supplementation of BMP-7 could improve the effect of TGF-β3 on chondrogenesis. The PLGA scaffold loaded with TGF-β3 and BMP-7 by the nanocomplex encapsulation method had a suitable morphology and beneficial controlled release properties for cartilage regeneration applications.

**Fig. 6:**
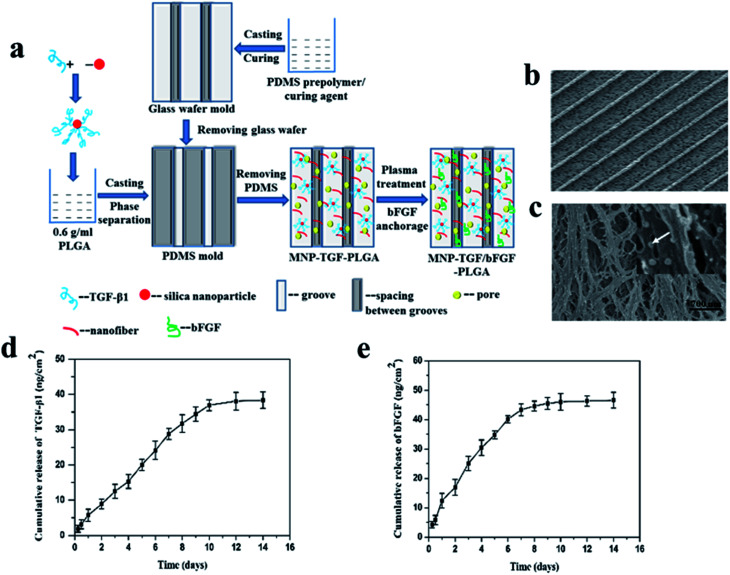
Schematic illustration of preparation (a) and morphology (b and c) of the MNP-TGF/bFGF-PLGA scaffold and cumulative releases of TGF-β1 (d) and bFGF (e) from the MNP-TGF/bFGF-PLGA scaffold.^93^

A certain amount of different growth factors are required at different stages of tissue regeneration and repair. So, it is necessary to use multiple methods to immobilize growth factors and make them release in different way. bFGF and TGF-β1 have been confirmed to be important growth factors that affect the phenotype and function of vascular smooth muscle cells. bFGF is an important angiogenic factor, which is a strong stimulating factor for the proliferation and migration of smooth muscle cell. TGF-β1 generally prevents vascular smooth muscle cell proliferation but promotes its migration and increases the production of extracellular matrix. The TGF-β1 and bFGF were simultaneously immobilized on the PLGA scaffold that had dual surface topographies of parallel arranged microgrooves and nanofiber structures respectively by encapsulating binding TGF-β1 silica nanoparticle and plasma treatment combined with bFGF anchorage methods (Fig. 6a–c).^93^ TGF-β1 was bound on silica nanoparticles (SiO_2_ NPs) through electrostatic effect and hydrogen bonding between TGF-β1 and the silica nanoparticle. The bound TGF-β1 on the SiO_2_ NPs distributed in the scaffold effectively avoid direct access of water, ensuring that the bound TGF-β1 exhibited a continuous release from the film for about 10 days and had almost no burst release (Fig. 6d). bFGF loaded on the PLGA scaffold by the technique of CO_2_ plasma treatment combined with bFGF anchorage could keep a pattern of continuous release for about 7 days after a moderate burst release, which was in accord with single CO_2_ plasma treatment combined with bFGF anchorage (Fig. 6e). The synergy effect of the dual surface topography and released growth factors endowed the PLGA scaffold with good capacity of regulating vSMC phenotype.

## 3. Conclusions

In short, growth factors can be loaded on the aliphatic polyester scaffolds during and after the preparation of scaffolds through direct blending or soaking, surface coating combined with covalent binding, plasma treatment combined with growth factor anchoring, micr-nano particle embedding, heparin binding, or a combination of multiple methods. The loading efficiency, releasing behavior and bioactivity of growth factor are different by using different methods (Table 1). Growth factors loaded on scaffolds could promote the growth and differentiation of relevant cells, repair and regeneration of defect tissues. Multiple growth factors relating to tissues repair are simultaneously loaded on scaffolds so that the growth factors are released at a certain concentration and time in different spaces maintaining biological activity. But it is still a great challenge to obtain scaffolds with same biological environment as damaged tissues. The influence of loading methods on the conformation of the growth factors and bioactivity still is unknown. The mechanisms of growth factor loading and release on aliphatic polyester scaffolds need to be further explored. The relationship between material, morphology, pore structure as well as surface properties of scaffolds and growth factor loading and release requires to be further clarified. With the in-depth research and discovery of the effects of various growth factors on the metabolism and growth of different cells and tissues, it is of great significance to further develop the methods of loading growth factors on aliphatic polyester scaffolds so as to more precisely control the growth factor release simulating the biological environment of damaged tissues. The growth factors precisely controlled by aliphatic polyester scaffold will be used in conjunction with the scaffolds and cells to repair and regenerate defect tissue.

**Table 1:** Release behavior and biological activity of growth factors loaded on different aliphatic polyester scaffolds by various methods

Scaffold	Loading method	Growth factor	Loading efficiency/capacity	*In vitro* release behavior	Biological activity assay		Application areas
					Cell test	Animal test	
PCL/PLGA6535/PLGA 8515 multilayered fibrous scaffold^32^	Directly blending	NT-3	—	Release of PLGA 6535 and PLGA 8515 reached a plateau after six weeks and eight weeks, respectively	—	Rat	Neural tissue regeneration
		BDNF					
		PDGF					
PLGA nanofiber scaffolds^34^	Directly blending	bFGF	54%	7 days (Group I), 14 days (Group II)	BMSCs	—	Bone regeneration
PLLA nanofiber scaffolds^35^	Directly blending	BMP-2	174 ng/implant	—	—	Rat	Bone regeneration
Bilayer scaffolds consisting of different PLGA nanofibers^36^	Directly blending	VEGF	57.27% of VEGF	Release of VEGF from the PLGA 50/50 layer was faster than that of PDGF from the PLGA 75/25 layer	HUVEC	—	Complex tissue engineering
		PDGF	51.76 %of PDGF				
Multi-layered PLLA nanosheets^37^	Directly dropping onto the middle of the PLLA nanosheet	rhFGF-2	—	—	—	Mouse	Bone regeneration
PLGA porous scaffold^89^	Directly soaking	bFGF	22.6%	A high initial burst and reached a standstill about 10 days	3T3 fibroblasts	Mouse	—
PLLA nanofibrous scaffold^33^	Directly blending (NGF) and soaking (VEGF)	NGF	—	Release rate of VEGF is higher (35.72 ± 0.29%) within 1 day than that of NGF (4.86 ± 1.00%). Release of VEGF reach a standstill (58.56 ± 1.31%) at the fourth day, but that of NGF reach a standstill (29.52 ± 0.91%) at the eleventh day	iPSCs-NCSCs	Rat	Peripheral nerve regeneration
		VEGF					
PLLA nanofibrous scaffold^42^	Surface collagen coating combined with covalent binding	TGF-β3	—	—	hBMSCs	—	Cartilage repair
PLGA/HA porous scaffolds^51^	PDA-mediated method	BMP-2	80% of IGF-1, 75% of BMP-2	After a burst release, the release subsequently slowed down with approximately 27% (IGF-1) and 39% (BMP-2) of the total growth factors released after 21 d	MC3T3-E1	Rabbit	Bone tissue engineering
		IGF-1					
3D-printed PLA scaffolds with interconnected microporous architectures^52^	PDA-mediated method	BMP-2	375.4 ng/scaffold	Sustained released of BMP-2 for up to 35 days	hMSCs	—	Bone tissue engineering
Tubular PCL scaffolds^53^	PDA-mediated method	VEGF	56.6 ng/scaffold	—	SMC EC	Rat	Vascular tissue engineering
PLLA nanofibrous scaffold^58^	Nanoparticle embedding	bFGF	48.71%	No burst release and a control release of nearly 30 days	C3H10T½ (C3) cells	Rat	Promotion of tendon healing
PLGA nanofibrous scaffold^62^	Microspheres embedding	VEGF	44.39%	Release last 20 days	HUVEC	Rat	Abdominal wall repair
PLLA nano-fibrous scaffolds^56,57^	PLGA microspheres with PDGF-BB or rhBMP-7 post-seeding	PDGF-BB rhBMP-7	77–93%	Temporally controlled fashion with prolonged duration and varying temporal patterns because of different PLGA nanosphere	Human gingival fibroblast	Rat	Complex tissue regeneration
PLGA films and porous scaffolds^40^	Plasma treatment combined with growth factor anchorage	bFGF	66.3%	Continuous release about 7 days after a moderate burst release	3T3 fibroblasts	—	Extensive tissue engineering
3D microtubule-orientated PLGA scaffold^78^	Plasma treatment combined with growth factor anchorage	bFGF	75.0%	Continuous release for 10 days after a moderate burst release	vSMC	—	Vascular tissue engineering
PLGA/PLLA microfiber scaffolds^88^	Heparin-mediated method	TGF-β1	26.8 ng/scaffold	Continuous release for 4 weeks after a moderate initial burst release	UCB-MSCs	Rabbit	Cartilage tissue engineering
H-PLGA/PLGA(70/30) porous scaffolds^89^	Heparin-mediated method	bFGF	71.3%	Slowly release and last over two weeks	3T3 fibroblasts	—	Extensive tissue engineering
PLLA nanofiber microspheres^91^	Heparin binding combined with nanospheres encapsulating	VEGF	—	Average burst release of VEGF on the first day was 20.5%, 54.7% was released within 1 week	HUVEC	Nude mouse	Pulp regeneration
				Release of VEGF consistently at a rate of approximately 1–2% per day for the last 3 weeks			
PLGA porous scaffold^92^	Heparin binding combined with nanospheres encapsulating	BMP-7	79% of TGF-β3	No burst and sustained release in a near zero-order kinetics for least 27 days	hMSCs	—	Cartilage regeneration
		TGF-β3	50% of BMP-7				
PLGA scaffold with parallel arranged microgrooves and nanofiber structures^93^	Nanoparticle binding (TGF-β1)	TGF-β1	—	Continuous release for about 10 days of TGF-β1	vSMC	—	Vascular tissue engineering
	Plasma treatment combined with anchorage (bFGF)	bFGF		Moderate burst release for bFGF and then about 7 days continuous release			

## Conflicts of interest

There are no conflicts to declare.

## Supplementary Material
